# Editorial: New challenges and future perspectives in brain imaging methods

**DOI:** 10.3389/fnins.2023.1265054

**Published:** 2023-11-01

**Authors:** Dahua Yu, Kai-Hsiang Chuang, Nico Sollmann

**Affiliations:** ^1^Inner Mongolia Key Laboratory of Pattern Recognition and Intelligent Image Processing, School of Information Engineering, Inner Mongolia University of Science and Technology, Baotou, China; ^2^Queensland Brain Institute, The University of Queensland, Brisbane, QLD, Australia; ^3^Department of Diagnostic and Interventional Radiology, University Hospital Ulm, Ulm, Germany; ^4^Department of Diagnostic and Interventional Neuroradiology, School of Medicine, Klinikum rechts der Isar, Technical University of Munich, Munich, Germany; ^5^TUM-Neuroimaging Center, Klinikum rechts der Isar, Technical University of Munich, Munich, Germany

**Keywords:** brain imaging methods, multi-modal imaging, magnetic resonance imaging, functional near-infrared spectroscopy (fNIRS), resting-state

Brain imaging has seen considerable advances over the recent years. Both developments and validation of advanced image acquisition techniques as well as post-processing and analyses pipelines have contributed to contemporary imaging, including parallel imaging, (semi-)automated segmentation, generation of synthetic images, and application of machine learning and radiomics. Multi-modal approaches using structural, metabolic, and functional imaging methods are emerging to build a framework for a better understanding of anatomical features and physiological processes of the brain. This Research Topic entitled “*New challenges and future perspectives in brain imaging methods*” included 12 articles (nine original research articles, one review article, one case report, and one brief research report) covering a broad spectrum of developments and applications in the field of advanced neuroimaging.

The majority of research articles published in this Research Topic covered advances in magnetic resonance imaging (MRI) (Tafuri et al.; Lan et al.; Alberti et al.; Tomasi and Volkow; Dai et al.; He et al.; Berson et al.; Ebersberger et al.; Charyasz et al.). In a multi-center study, Tafuri et al. evaluated harmonization of radiomics features to limit site-dependent effects, given that radiomics features commonly demonstrate considerable variability related to variations between sites and imaging protocols. To overcome this issue that is limiting the reproducibility and generalizability of radiomics, T1-weighted (T1w) sequences from different sites and derived from healthy subjects and patients with Parkinson's disease (PD) were analyzed, and the authors found that data from healthy subjects may be corrected with a ComBat-based harmonization approach (Tafuri et al.). Further, an area under the curve (AUC) of 0.77 (harmonized radiomics features) vs. an AUC of 0.71 (raw radiomics measures) was revealed to distinguish patients with PD from healthy subjects, emphasizing the considerable impact of site-related effects that need to be addressed by harmonization approaches also in future multi-centric studies (Tafuri et al.). A study by Lan et al. aimed to investigate the role of extracellular fluid in the context of cerebral small vessel disease (CSVD), considering a multi-sequence MRI protocol that has been composed of T1w and T2-weighted (T2w) imaging including fluid-attenuated inversion recovery (FLAIR) sequences, as well as diffusion tensor imaging (DTI) and susceptibility-weighted imaging (SWI). White matter (WM) hyperintensities were identified on T2w images (using Fazekas scoring), cerebral microbleeds were assessed on SWI, perivascular spaces (using 4-point scoring) were screened on T2w images, and lacunar infarction was detected on T2w and T1w images, in order to generate a CSVD MR marker score (Lan et al.). Furthermore, mean free water (FW) and fractional anisotropy (FA) were calculated based on DTI data for each patient's WM mask (Lan et al.). The authors found that mean FW of WM was significantly associated with some of the CSVD MR markers, and age, hypertension, diabetes mellitus, and FW were significantly associated with the derived CSVD MR marker score, while FW of WM matter was significantly inversely associated with FA (Lan et al.). Hence, an MRI-derived scoring system may be helpful to systematically assess CSVD burden, and extracellular fluid of WM may relate to CSVD severity and decline in WM integrity (Lan et al.). The DTI technique with extraction of FA has also been used by Alberti et al. to investigate varicella zoster-related brachial plexopathy in a case report of a 72-year-old woman, who presented with unilateral segmental paresis of the upper limb and painful dermatomal vesicular eruption. The authors observed a decrease in FA and an increase in mean, axial, and radial diffusivity for C6 and C7 roots of the affected compared to the unaffected side, indicative of microstructural fiber damage, which could be quantified by the presented approach and then considered during initial assessment and follow-up investigations in patients with plexopathy (Alberti et al.).

Using functional MRI (fMRI), Tomasi and Volkow evaluated the impact of head motion during image acquisition on functional connectivity (FC) by applying connectome-based predictive modeling to fMRI data with low frame-to-frame motion. Leave-one-out was used for internal cross-validation of head motion prediction in half of the dataset, and two-fold cross-validation was used in the other half (defined as the independent sample) (Tomasi and Volkow). Both parametric testing and connectome-based predictive modeling permutations for null hypothesis testing demonstrated strong linear associations between observed and predicted head motion (Tomasi and Volkow). Of note, prediction accuracy for head motion was higher for task-based fMRI as compared to rest-fMRI (whereas, however, prediction accuracy was lower for individuals with low motion than for those with moderate motion), and denoising attenuated the predictability of head motion, while stricter framewise displacement thresholds for motion censoring did not alter the accuracy of the predictions obtained with lenient censoring (Tomasi and Volkow). In a study by Dai et al., resting-state fMRI was used to define reproducible resting-state networks (RSNs) in healthy rats, followed by investigations of FC changes within and between RSNs following a chronic restraint stress model, given that no consensus has been made available for reproducible RSNs in rodents. In the anesthetized animals, default mode network-like, spatial attention-limbic, corpus striatum, and autonomic network patterns were identified (Dai et al.). The chronic restraint stress model decreased the anti-correlation between default mode network-like and autonomic networks and the correlation between amygdala and the nucleus accumbens and ventral pallidum within the corpus striatum network, while high individual variability in FC prior and subsequent to the stress model within RSNs became evident (Dai et al.). This high variability in FC may imply that rats may demonstrate different neural phenotypes, which should be classified further in the future to improve the sensitivity and translational impact of rodent models (Dai et al.). Furthermore, one study applied perfusion imaging by means of dynamic susceptibility contrast (DSC)-MRI in patients who had suffered ischemic stroke within the internal carotid artery territory, had related neurological deficits for at least one week after the insult, and did not receive intravenous thrombolysis or mechanical thrombectomy (He et al.). The patients underwent perfusion MRI at four time points (few weeks to 7 months after ischemic stroke), together with assessments of the National Institutes of Health Stroke Scale (NIHSS) and the modified Rankin Scale (mRS) (He et al.). Following the analysis of perfusion MRI with a whole-lesion approach and voxel-based parametric response mapping at each time point, voxels with decreased time-to-maximum values, and voxels with decreased or increased relative cerebral blood volume values derived from assessments about 3 months after ischemic stroke were superior to the mean values of the corresponding maps when predicting long-term clinical outcome at 7-months follow-up investigations (He et al.). Further, correlations were identified between the clinical prognosis and DSC-MRI parameters, thus potentially emphasizing the superiority of parametric response mapping over the whole-lesion approach for predicting long-term clinical outcome, which could be accompanied by information on heterogeneity of stroke lesions that can be derived from parametric response mapping (He et al.).

Regarding MRI with non-standard field strength, studies on both low- and high-field imaging have been published in the Research Topic (Berson et al.; Ebersberger et al.; Charyasz et al.). Specifically, Berson et al. used a point-of-care 1-Tesla MRI system to image intracranial pathologies within neonatal intensive care units (average gestational age at scan time: 38.5 ± 2.3 weeks) by acquiring T1w, T2w, and diffusion-weighted sequences. Transcranial ultrasound, 3-Tesla MRI, or both were available for comparison among the majority of the examined subjects, with the most common indications for 1-Tesla MRI being term-corrected age scans for extremely preterm neonates, intra-ventricular hemorrhage follow-up examinations, and suspected hypoxic injury (Berson et al.). The 1-Tesla MRI scan enabled identification of ischemic lesions in two infants with suspected hypoxic injury, confirmed by follow-up 3-Tesla MRI, while two lesions were identified on 3-Tesla MRI that were missed on 1-Tesla MRI (punctate parenchymal injury vs. microhemorrhage and small intra-ventricular hemorrhage) (Berson et al.). On the other hand, 1-Tesla MRI enabled the detection of parenchymal microhemorrhages that were not captured by ultrasound, emphasizing the potential of a point-of-care 1-Tesla MRI system on a neonatal intensive care unit (Berson et al.). Using a 7-Tesla MRI system in a proof-of-concept study, Ebersberger et al. investigated a 55-year-old male patient with early subacute stroke by dynamic oxygen-17 imaging in the context of a three-phase inhalation experiment, thus enabling direct and non-invasive assessment of cerebral oxygen metabolism. However, the analysis of relative oxygen-17 water signal for the stroke region compared to the healthy contralateral side revealed no significant difference; nevertheless, the technical feasibility of oxygen-17 imaging has been demonstrated in this study, which could in the future help to distinguish between viable and non-viable tissue in neurovascular diseases (Ebersberger et al.). Furthermore, in a study by Charyasz et al., a 9.4-Tesla MRI system was used for dedicated functional mapping of sensorimotor activation in the human thalamus within the scope of a task-based fMRI experiment with assessment of subject-specific sensorimotor blood oxygenation level dependent (BOLD) responses from a combined passive sensory (tactile finger) and active motor (finger tapping) paradigm. Both applied tasks related to an increase in the BOLD signal response in the lateral nuclei and pulvinar nuclei group, with the finger tapping paradigm resulting in a stronger BOLD response compared to the tactile finger paradigm and also engaging the intralaminar nuclei group (Charyasz et al.). This study with high-field imaging may give detailed insight into functions of individual thalamic nuclei regarding processing of input signals from the sensorimotor domain (Charyasz et al.).

Besides MRI, studies on other techniques such as electrocorticography (ECoG), contrast-enhanced electrical impedance tomography (C-EIT), and functional near-infrared spectroscopy (fNIRS) have been considered in the Research Topic (Kentar et al.; Zhang et al.; Ye et al.). In this regard, ECoG was used by Kentar et al. to study spatial and temporal signal changes following ischemia due to clipping of the middle cerebral artery (for 8–12 min) in a pig model. During the experiments, five-contact ECoG stripes were placed bilaterally over the fronto-parietal cortices, thus corresponding to the irrigation territory of the middle as well as anterior cerebral artery, with ECoG recordings obtained before and after the occlusion (Kentar et al.). The electrodes close to the occlusion showed instant decay in all frequency bands and spreading depolarization onset during the first 5 h, whereas electrodes far from the occlusion showed immediate loss of fast frequencies and progressive decline of slow frequencies with an increased spreading depolarization incidence between 6 and 14 h (Kentar et al.). After 8 h, the electrode capturing the anterior cerebral artery territory showed secondary reductions of all frequency bands except gamma and high spreading depolarization incidence within 12 to 17 h (Kentar et al.). Furthermore, all electrodes showed a decline in all frequency bands during spreading depolarization, while after spreading depolarization passage the frequency band recovery was impaired only in electrodes for the middle cerebral artery; thus, ECoG may allow to capture and characterize infarct progression and secondary brain injury (Kentar et al.). In a pilot study using a rabbit model, Zhang et al. aimed to assess the feasibility of monitoring perfusion with the C-EIT technique. The animals were allocated to a group without and with unilateral internal carotid artery occlusion due to clipping (for 30 min), following injection of glucose as an electrical impedance-enhanced contrast agent through the right internal carotid artery (Zhang et al.). In the non-occlusion group, impedance values of the left cerebral hemisphere did not change significantly, whereas the impedance value of the right cerebral hemisphere gradually increased (Zhang et al.). In the occlusion group, the impedance values of both cerebral hemispheres increased gradually and then began to decrease after reaching the peak value, with significant differences in the remodeling impedance values between the left and right hemispheres in both groups, respectively, while also a significant difference for the left hemispheres between groups was revealed (Zhang et al.). Hence, C-EIT may determine brain perfusion, offering broad application perspectives from disease progression monitoring or collateral circulation judgment to detections of malignancy-related lesions (Zhang et al.). Finally, a review article by Ye et al. set the focus on recent hotspots and trends in fNIRS research, highlighting that major topics of interest included activation, prefrontal cortex, working memory, cortex, and fMRI, while particularly research on gait function has received much attention. According to the review, fNIRS seems to play an increasingly important role as a non-invasive brain functional imaging technique for the detection of function-related activity (Ye et al.).

To conclude, this Research Topic covered a large spectrum of advanced imaging technology, with a focus of the published articles on applications of MRI. Both human and animal studies have been considered, contributing to an improved understanding of brain (patho)physiology, image acquisition and processing strategies, and disease characteristics. However, also techniques that are not widely available or are emerging have been covered, which could provide either unique or complementary information when related to MRI investigations. Finally, more research is necessary regarding tailoring existing procedures to clinical needs for methods that have already been made available for the *in-vivo* study of the human brain, or to accelerate the transition of findings from animal studies to potential human applications.

## Author contributions

DY: Writing—original draft. K-HC: Writing—review and editing. NS: Writing—review and editing.

